# A new technique for separating radioulnar synostosis with vascularized flap: a case report

**DOI:** 10.1016/j.jseint.2023.03.016

**Published:** 2023-04-15

**Authors:** Yi-Chih Chen, Ken N. Kuo, Takehiko Takagi, Shinichiro Takayama, Po-Jen Shih, Chia-Hsieh Chang

**Affiliations:** a Department of Biomedical Engineering, National Taiwan University, Taipei, Taiwan; b Department of Orthopaedics, Cathay General Hospital, Taipei, Taiwan; c Department of Orthopaedic Surgery, National Taiwan University Hospital, Taipei, Taiwan; d Cochrane Taiwan, Taipei Medical University, Taipei, Taiwan; e Division of Orthopaedic Surgery, Department of Surgical Specialties, National Center for Child Health and Development, Setagaya-Ku, Tokyo, Japan; f Department of Pediatric Orthopaedics, Chang Gung Memorial Hospital, Taoyuan, Taiwan

**Keywords:** Radioulnar synostosis, New technique, Biceps transfer, Adipofascial flap, Osteotomy, Recurrent synostosis

Congenital radioulnar synostosis is a rare condition occurring in both boys and girls and is characterized by an abnormal connection between the radius and the ulna. The synostosis commonly occurs in the proximal one-third of the forearm and restricts forearm movements in pronation. Approximately 20% of cases have a family history of the congenital disorder.^1^^,^^7^^,^^11^ The majority of patients have difficulties performing daily activities, such as maintaining hygiene care, eating with chopsticks, answering the phone, and combing their hair.^2^^,^^7^

Treatment of congenital radioulnar synostosis aims to improve functionality and convenience for patients when performing daily activities by reposition of forearm rotation or increasing the range of forearm rotation. Derotational osteotomy is a reliable procedure that fixes the forearm in a functional position. Horii et al^4^ reported that a single osteotomy at the radial diaphysis was effective in correcting pronation deformity, but the forearm motion remained restricted after osteotomy. Surgical separation of the synostosis often results in recurrence and motion limitation; therefore, several methods have been proposed to prevent re-synostosis. Yabe et al interposed the anconeus muscle between the bones to prevent re-synostosis. However, Miura et al reported that all cases treated using Yabe’s method experienced re-synostosis.^9^^,^^12^ Kanaya et al^6^ therefore suggested inserting a free vascularized adipofascial graft to prevent re-synostosis. However, free vascularized fascio-fat grafting requires microvascular anastomosis, which is technically demanding. In addition, proximal radius osteotomy carries the risk of the posterior interosseous nerve injury. Given these surgical constraints, we modified Kanaya’s method by inserting the adipofascial flap with the feeding branch of the posterior interosseous artery^3^ and performing an osteotomy at the middle third of the radius.^4^ This case report describes the technical details and results of a modified method for treating congenital radioulnar synostosis.

## Case report

**Figure 1:**
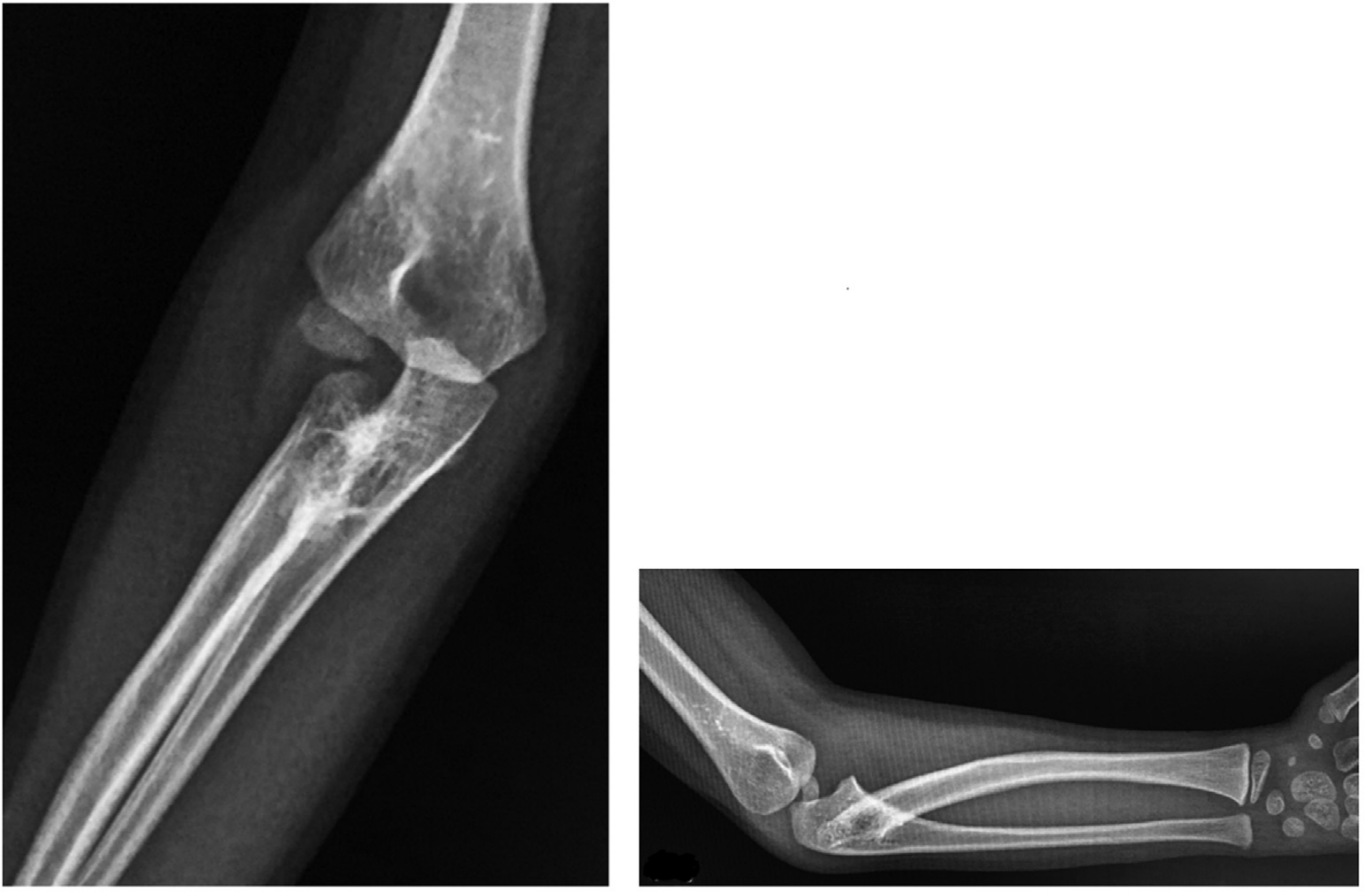
Radiographs of the right elbow showed proximal radioulnar synostosis, posterior radial head dislocation, and posterior bowing of the radius.

A 6-year-old boy presented to our orthopedic clinic with a primary complaint of restricted right forearm rotation. The motion limitation was first observed at the age of 2. Physical examination revealed that the forearm was ankylosed in 20° of pronation without any supination motion. The elbow had flexion-extension motion from 135° to 0°. Plain radiographs of the right elbow revealed proximal radioulnar synostosis with posterior radial head dislocation (Fig. 1).

**Figure 2:**
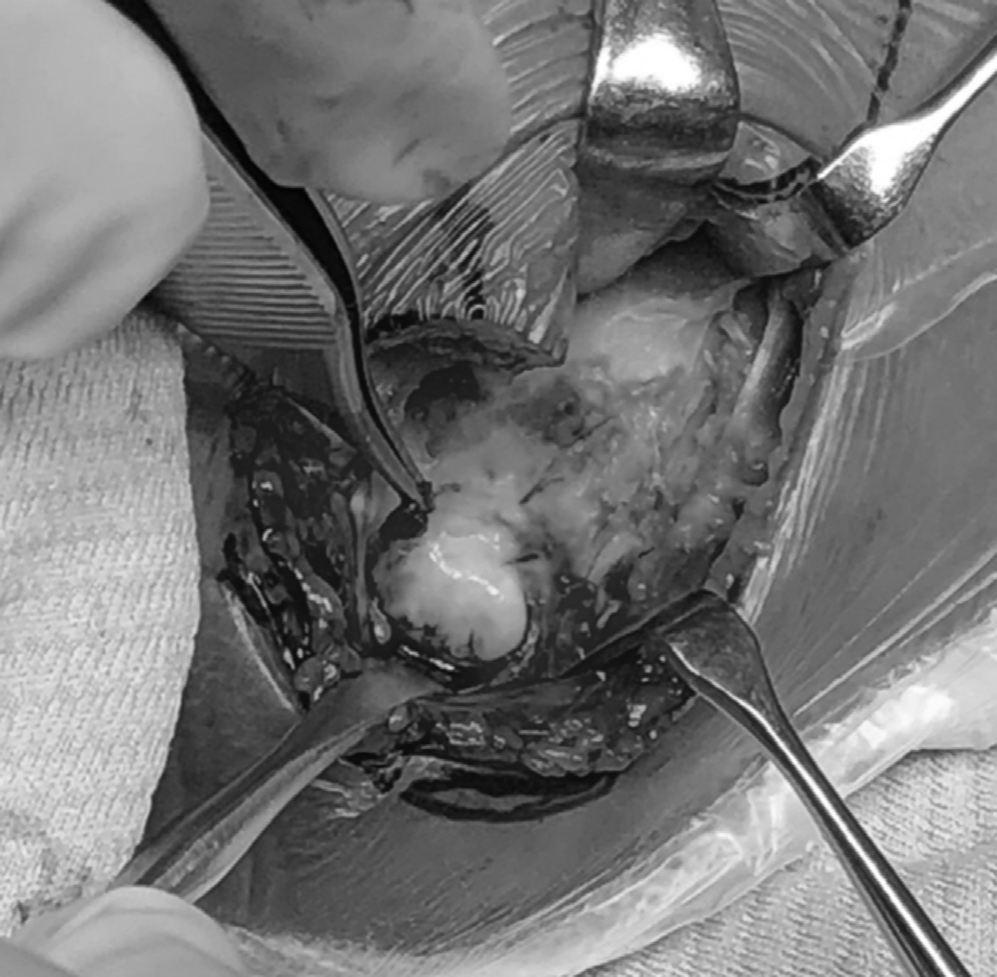
The radial head was hypoplastic, posteriorly dislocated without concave articular surface.

**Figure 3:**
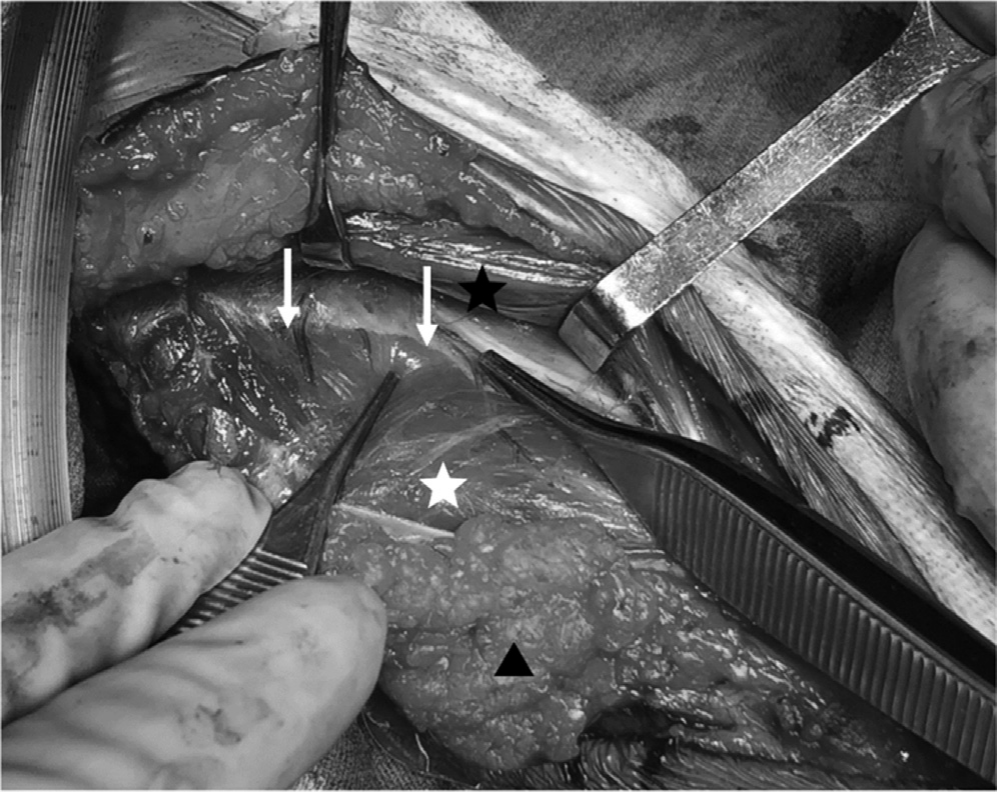
A vascularized adipofascial graft by the posterior interosseous artery was inserted at the separated synostosis site. The donor site of a fat graft (black arrowheads), posterior interosseous artery perforators (white arrow), and extensor carpi ulnaris (white asterisk) and extensor digitorum (black asterisk) are presented.

**Figure 4:**
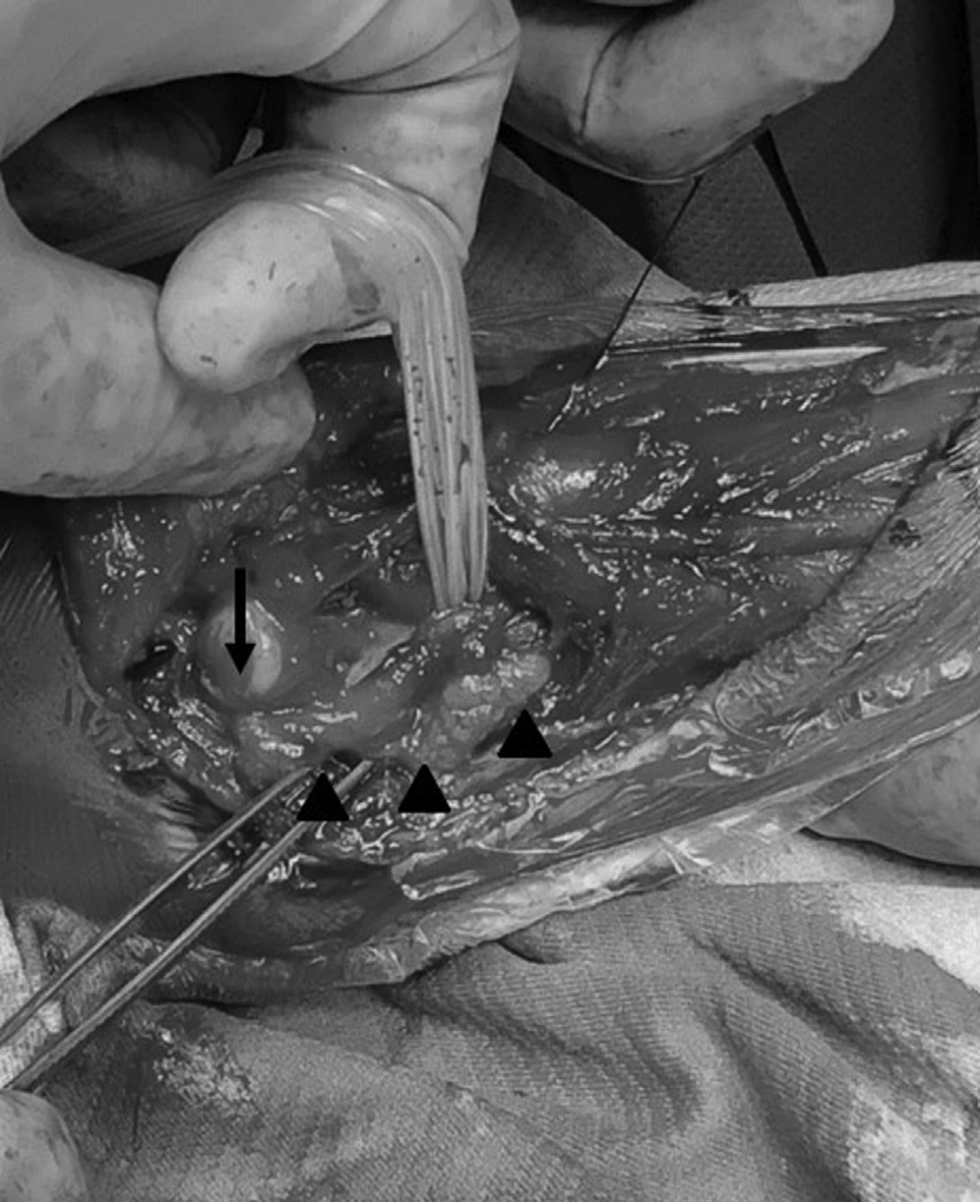
The graft was placed between the separated radius and ulna. The radial head (arrow) and adipofascial graft (arrowheads) are shown.

The boy received surgical treatment at the age of 6 years. A posterolateral approach (Kocher approach) to the elbow was used to examine the synostosis and synchondrosis. Under direct observation, the radial head was hypoplastic, and was posteriorly dislocated without a concave articular surface (Fig. 2). The proximal radius and ulna were then separated by resecting the synchondrosis along the radial head cartilage and synostosis along the proximal radial metaphysis. The biceps brachii tendon inserted on the ulna instead of the radial tuberosity. In order to improve supination power, the biceps tendon was detached from the ulna and transferred to the radial neck. The surgical incision was extended to the distal two-thirds of the forearm to examine the posterior interosseous artery between the extensor carpi ulnaris and the extensor digitorum (Fig. 3). A vascularized adipofascial mass (6 cm × 7 cm × 1.5 cm in size) was raised with a proximal pedicle. To address radioulnar synostosis with posterior dislocation of the radial head, we performed a wedge osteotomy with 10-mm shortening at the middle radius along with 90° of external rotation of the distal radius. The osteotomy site was fixed with a titanium locking plate. The vascularized adipofascial flap was inserted at the synostosis site between the radius and ulna in a posterior to anterior direction (Fig. 4).

**Figure 5:**
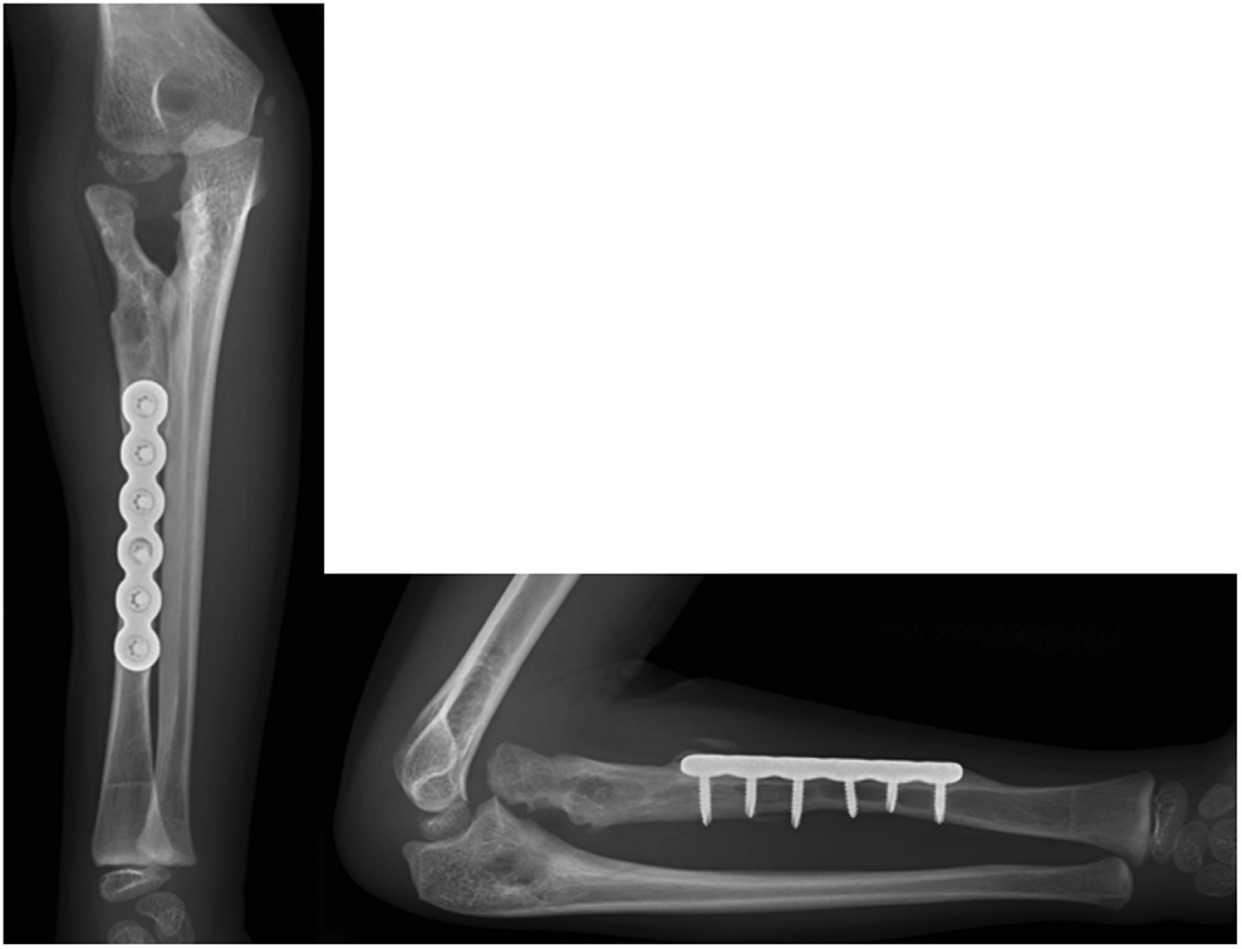
Radiographs 1 year postoperatively showing that the synostosis space remained separated but the radial head was anteriorly dislocated.

**Figure 6:**
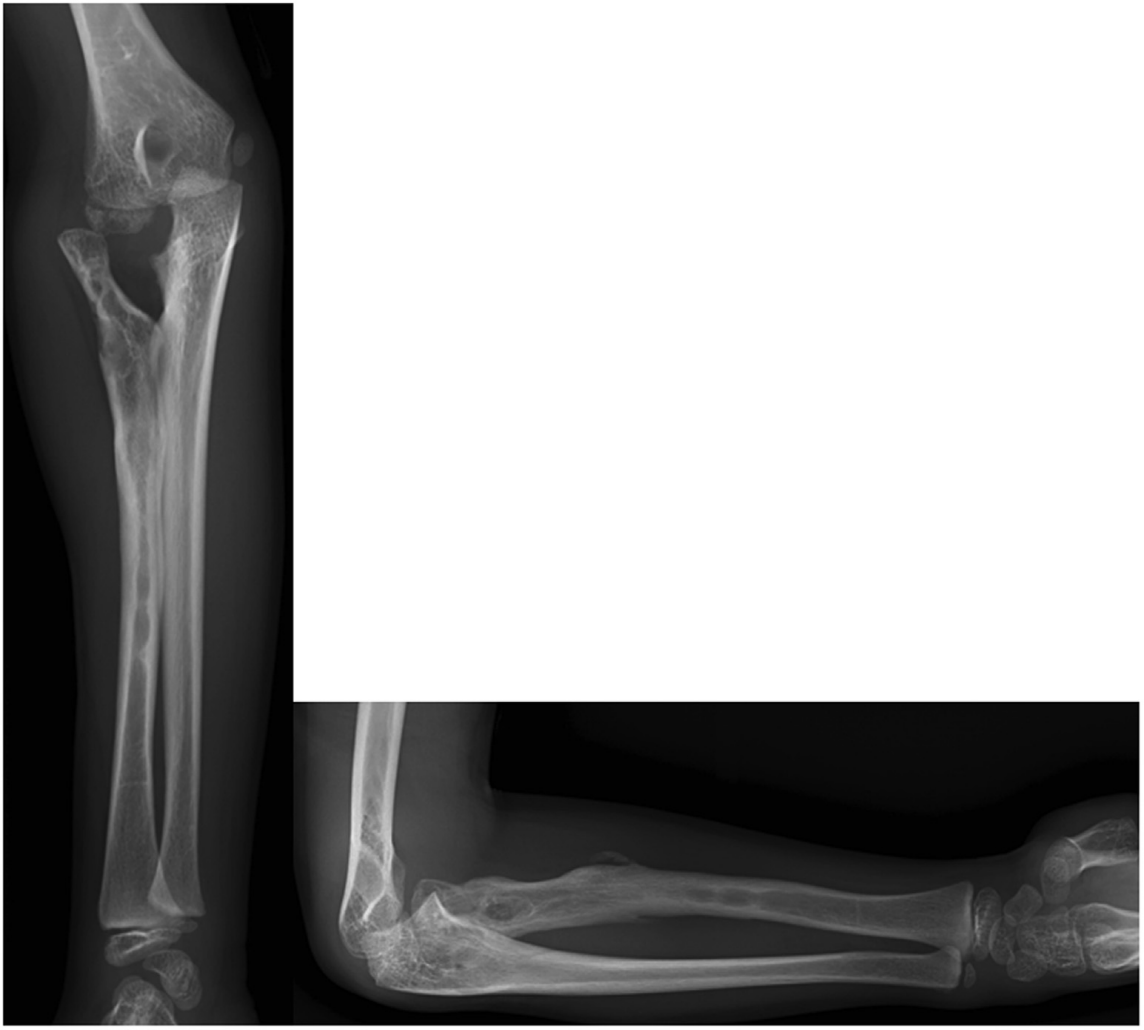
Radiographs 2 years postoperatively. The radius and ulna were separated and the radial head anterior dislocation persisted.

**Figure 7:**
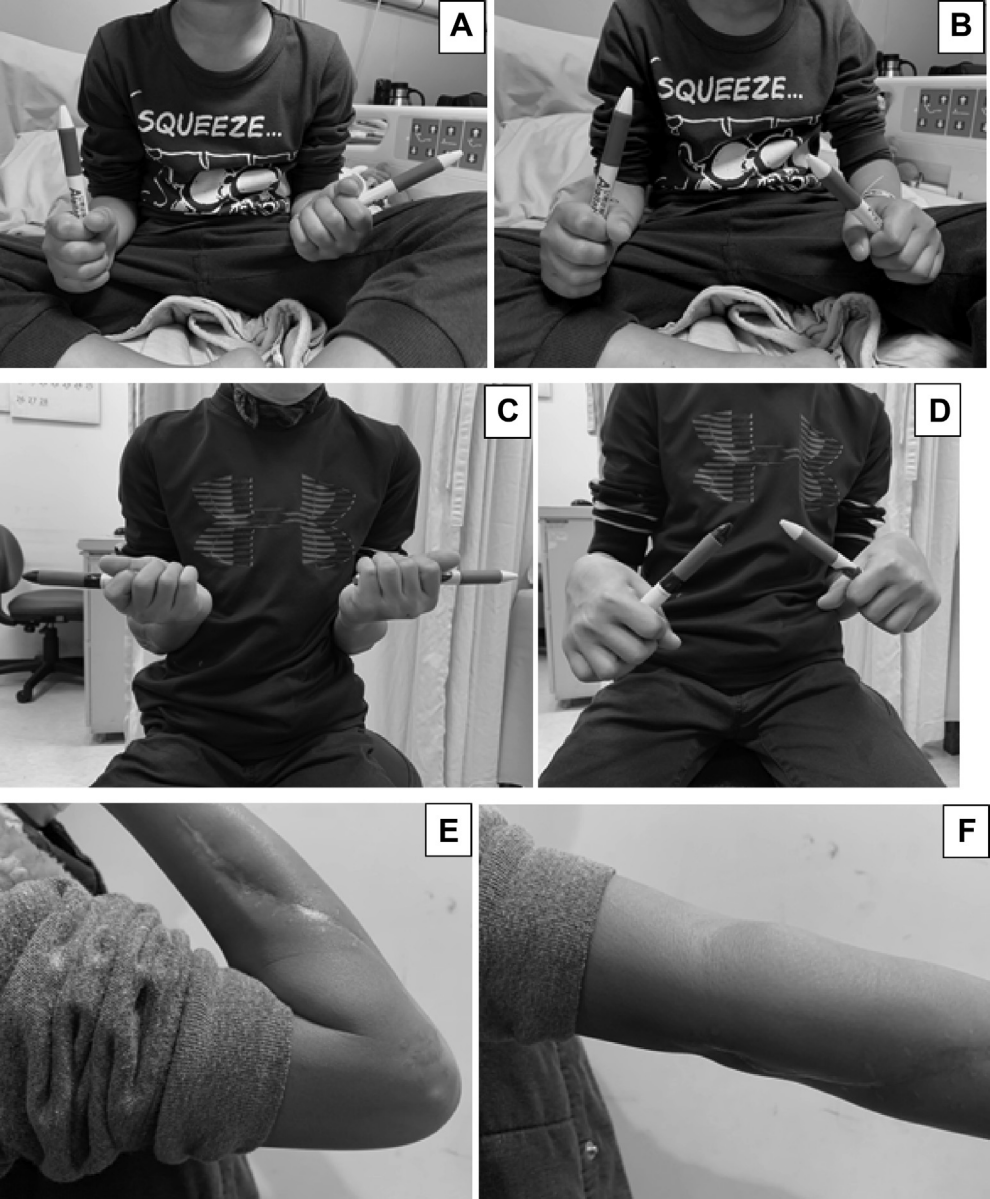
Clinical photographs showing the boy with right forearm fixed in 20° of pronation preoperatively (**A** and **B**). At 2 years postoperatively, the ranges of motion were forearm supination 90° (**C**), pronation 40° (**D**), elbow flexion 135° (**E**), and extension 0° (**F**).

Postoperatively, a long arm splint was used to keep the elbow in 90° of flexion and the forearm in full supination for 3 weeks. After splint removal, the rehabilitation program started with range of motion exercise of forearm pronation and supination, in addition, a special exercise of elbow flexion in supination to elbow extension in pronation was carried out. Two months after surgery, the range of the forearm rotation improved to 85° of supination and 35° of pronation. The osteotomy site was healed at 3 months postoperatively. The forearm pronation and supination further improved to as the uninvolved side 6 months postoperatively. Plain radiographs revealed that the radius and ulna bones were maintained separated and the radial head was anteriorly dislocated. The implants at the middle radius were removed without extending surgical dissection to check the fat graft 1 year postoperatively (Fig. 5). Radiography 2 years postoperatively showed the radius and the ulna were still separated. A radiolucent shadow that corresponds to the pedicled fat was recognizable between the proximal radius and the ulna (Fig. 6). The ranges of motion were forearm supination 90°, pronation 40°, elbow flexion 135°, and extension 0° (Fig. 7). He had no limitation in daily activities but used shoulder abduction to increase pronation. The boy and his parents were satisfied with the improvement in forearm rotation and function.

## Discussion

The goals of treatment for congenital radioulnar synostosis are to improve patients’ self-care abilities and quality of life by restoring forearm rotation. We treated the radioulnar synostosis in a 6-year-old boy by the placement of a vascularized pedicle adipofascial flap with posterior interosseous vessels that prevented re-synostosis without the necessity of technically demanding microvascular anastomosis. The forearm supination was directly improved by derotation osteotomy at the middle third of the radius to prevent posterior interosseous nerve injury that commonly occurred in osteotomy at the proximal radius.

Recurrent synostosis is related to the failure of separating radioulnar synostosis. To prevent recurrence, Kanaya and Ibaraki used a free vascularized fascio-fat graft between the radius and the ulna.^6^ Kawaguchi et al^8^ employed the same method and reported good results without developing recurrent ankyloses. However, free vascularized grafting requires microsurgery to rebuild vascular circulation, and the vessels to a fat pad are tiny and fragile. Therefore, we used a regional pedicle flap instead for its technically simpler approach as well as the original and reliable blood supply.

Derotational osteotomy can be used to correct forearm position and radial head dislocation. Kanaya et al^5^ performed the osteotomy at the proximal radius near the radial head. However, the plate fixation on the proximal radius is often unstable due to a short proximal segment, and the proximal radius osteotomy may cause injury to the posterior interosseous nerve. Given these concerns, Sakamoto et al^10^ modified Kanaya’s method by performing an osteotomy at the middle radius for children with posterior dislocation of the radial head. We agreed with the use of Sakamoto's radial radii analysis for different types of radial head dislocation. Therefore, we performed the derotation osteotomy at the middle third of the radial diaphysis to correct forearm pronation position and reduced the posterior radial head dislocation. Although the anterior dislocation of the radial head is present in this case because of hypoplasia of the radial head, the patient can still rotate his forearm and perform self-feeding and personal hygiene without any restrictions.

In this case the radial head was hypoplastic and posteriorly dislocated. However, acquired radial head dislocation at a young age and post-traumatic synostosis or myositis ossificans that blocked forearm rotation cannot be completely ruled out. Intraoperatively, the radial head was hypoplastic without concavity for articulation with the capitellum. The pathology increased risks of re-dislocation of the radial head.

## Conclusion

We report a new surgical technique including a regional vascularized pedicle adipofascial flap to prevent re-synostosis and radial osteotomy to correct forearm position and increase supination movement. The preliminary results in restoring forearm supination and improving daily activity function are encouraging. Further study including more surgical cases and longer follow-up is required.

## Disclaimers

Funding: No funding was disclosed by the authors.

Conflicts of interest: The authors, their immediate families, and any research foundation with which they are affiliated have not received any financial payments or other benefits from any commercial entity related to the subject of this article.

Patient consent: Obtained.
